# Computer-assisted syllable analysis of continuous speech as a measure of child speech disordera)

**DOI:** 10.1121/10.0028176

**Published:** 2024-08-19

**Authors:** Marisha L Speights, Joel MacAuslan, Suzanne Boyce

**Affiliations:** 1 Department of Communication Sciences and Disorders, Northwestern University, Evanston, Illinois 60202, USA; 2 Speech Technology and Applied Research Corporation, Lexington, Massachusetts 02421, USA; 3 Department of Communication Sciences and Disorders, University of Cincinnati, Cincinnati, Ohio 45219, USA

## Abstract

In this study, a computer-driven, phoneme-agnostic method was explored for assessing speech disorders (SDs) in children, bypassing traditional labor-intensive phonetic transcription. Using the SpeechMark^®^ automatic syllabic cluster (SC) analysis, which detects sequences of acoustic features that characterize well-formed syllables, 1952 American English utterances of 60 preschoolers were analyzed [16 with speech disorder present (SD-P) and 44 with speech disorder not present (SD-NP)] from two dialectal areas. A four-factor regression analysis evaluated the robustness of seven automated measures produced by SpeechMark^®^ and their interactions. SCs significantly predicted SD status (*p* < 0.001). A secondary analysis using a generalized linear model with a negative binomial distribution evaluated the number of SCs produced by the groups. Results highlighted that children with SD-P produced fewer well-formed clusters [incidence rate ratio (IRR) = 0.8116, *p* ≤ 0.0137]. The interaction between speech group and age indicated that the effect of age on syllable count was more pronounced in children with SD-P (IRR = 1.0451, *p* = 0.0251), suggesting that even small changes in age can have a significant effect on SCs. In conclusion, speech status significantly influences the degree to which preschool children produce acoustically well-formed SCs, suggesting the potential for SCs to be speech biomarkers for SD in preschoolers.

## I. INTRODUCTION

In the course of development, children become more and more facile at voluntary coordination of the motoric movements necessary for producing complex syllables (Greenberg, 1998; MacNeilage, 1998; Oller *et al.*, 1999). The mastery of syllable production and, in particular, complex syllable production, has been revealed to be a powerful predictor of later communication skills (Aram and Hall, 1989; Catts, 1993; Ingram, 2002; Masso *et al.*, 2016; Oller *et al.*, 2010; Stoel-Gammon, 2010; Young, 1991). It has long been reported that children with delayed speech acquisition show a slower trajectory for complex syllable production, and many authors have suggested that such deviations in the trajectory of syllable acquisition may serve as a diagnostic marker of future speech delay (Fell *et al.*, 1999; Fell *et al.*, 2002; Flipsen, 2006b; Oller *et al.*, 2010; Xu *et al.*, 2014).

The traditional clinical encounter represents a delicate balance between the need for accurate classification of children's speech disorder (SD) and the need for efficient use of time. It is acknowledged that the most sensitive and realistic picture of child speech production is gained by analyzing continuous, relatively long, and spontaneous stretches of speech, but there are few clinical tools that can maximize accuracy (Klein, 1981; Klein and Liu-Shea, 2009; Lagerberg *et al.*, 2014; Morrison and Shriberg, 1992; Paul and Jennings, 1992) while also achieving efficient use of clinician time (Bleile, 2002). Traditionally, broad phonetic transcription has been one of the central tools used for capturing and measuring production accuracy in speech samples for the purpose of classifying SDs (Kent, 1996; Stoel-Gammon, 2001). However, broad transcription of large stretches of speech is labor-intensive. Further, it is most useful when the transcriber knows what words or phrases were intended and the transcription of unintelligible segments in continuous speech becomes increasingly unreliable and takes more time as listeners attempt to parse the speech stream into transcribable units. This problem is even more acute when the context of the conversation is unknown (Flipsen, 2006b). Clinicians with specialized training using narrow transcription can provide a finer grade of analysis, but this is seldom performed because of the cost of increased transcription time and potential for error (Ball and Rahilly, 2002; Shriberg and Lof, 1991). Alternatively, some researchers have turned to the analysis of syllables rather than phonetically transcribing continuous speech samples for young children to address the analysis when speech includes unintelligible segments (Flipsen, 2006a; Lagerberg *et al.*, 2014; Stoel-Gammon, 2010).

Syllables are a universal unit of speech across world languages and play a critical role in speech articulation and perception (Gómez *et al.*, 2014; Shattuck-Hufnagel, 2011; Sun and Poeppel, 2023; Tuller and Kelso, 1991). Because they are organized as a string of consonants and vowels according to an increasing/decreasing pattern of sonority, the clustering of acoustic features characteristic of syllable shape can be reliably detected in the acoustic signal. Thus, detecting the structure of this unit allows for language-independent analysis. Additionally, because syllables are phoneme-agnostic (i.e., a particular syllable shape, such as CCVCC, may describe different combinations of consonants and vowels), a syllable-focused analysis does not require the speech signal to be perfectly intelligible (Flipsen, 2006a). Languages differ in terms of which syllable structures are valid; for English, multiple researchers have documented the average number and typical structure of syllables in spontaneous speech throughout various developmental stages (MacNeilage, 1998; Oller *et al.*, 1999; Oller *et al.*, 2010).

As the above studies demonstrate, the typical time course of speech production maturity has the following characteristics. First, as they age, children typically produce longer utterances. Second, those longer utterances tend to show more successfully uttered complex syllables. Third, children produce the same sequence of phonemes—that is, syllable, word, and sentence combinations—with shorter and shorter durations (Namasivayam *et al.*, 2020). At the same age, children with speech disorder present (SD-P) often produce shorter utterances and less complex syllables (Flipsen, 2006b; Lee *et al.*, 1999). Accordingly, the cumulative evidence from these studies indicates that at least for children speaking English, there is a developmental progression characterized by the use of longer words, an increase in the length of utterances, and an increase in the average number of syllables per word as a function of age. Studies have shown that children with lower speech production capacity resemble younger children on these variables and the tendency to produce simple syllabic structure even when attempting to produce more complex syllables (Flipsen, 2006b; Xu *et al.*, 2014). As speech production capacity improves, there is an increase in the complexity of syllables uttered (Stoel-Gammon, 2010). Studies have also found a trend for children with SD-P to show longer segment durations (Flipsen, 2002). These aspects of children's speech may mean that children with reduced speech production capacity simplify the task of producing complex speech sequences in several ways by: (1) breaking longer linguistic sequences into shorter stretches of speech, (2) simplifying complex syllables into simpler syllables, (3) lengthening the period of time over which speech sequences are produced, and (4) slowing overall speech rate (Namasivayam *et al.*, 2020). Children with SD-P may have more speech motor variability, resulting in poorer accuracy and longer movement duration (Flipsen, 2002; Kent and Forner, 1980; Kim and Stoel-Gammon, 2010; Shriberg *et al.*, 2010; Terband *et al.*, 2011), such as those commonly observed in young children as they overcome syllable structure constraints (Flipsen, 2002; Stoel-Gammon, 1987; Tanner Dyson, 1988). Accordingly, these aspects can be used to track speech motor development as the speech system matures (MacNeilage, 1998; Oller *et al.*, 2010).

Technological advancements in signal processing, pattern recognition, and computational algorithms have expanded the capabilities for the analysis of larger corpora by automating syllable segmentation in adult speech (Mermelstein, 1975; Seshadri and Rasanen, 2019; Shankar and Venkataraman, 2019; Villing *et al.*, 2005). One approach has been to employ automatic speech recognition (ASR) syllable-based recognizers for the analysis of continuous speech (Chen, 2011; Ganapathiraju *et al.*, 1997; He *et al.*, 2018; Hunt *et al.*, 1980; Panda and Nayak, 2016; Sethy *et al.*, 2006; Wu *et al.*, 1997; Yarra *et al.*, 2016). However, ASR-based methods resemble traditional methods in that syllable structure is determined based on phonemic transcription; after a word is recognized, it is broken down as a sequence of phonemes, and the syllabic structure of the word is identified from that sequence. In addition, performance is dependent on the existence of a large training database of speech that includes the annotated transcription of words. Because the speech of young children is often unintelligible, it is hard to be sure which words were uttered. Accordingly, the application of ASR methods to the speech of young children has been limited (Booth *et al.*, 2020; Fernando *et al.*, 2016; Shivakumar *et al.*, 2014; Shivakumar and Georgiou, 2020; Yeung and Alwan, 2018). Only a few studies have attempted automatic measurement of syllable patterns in a phoneme-agnostic way and even fewer have focused on automated measurements or continuous vocalizations (Fell *et al.*, 1999; Fell *et al.*, 2002; Lau *et al.*, 2023; Oller *et al.*, 2010; Speights Atkins *et al.*, 2019).

To overcome these constraints, we take a phoneme-agnostic approach to identifying and classifying syllabic sequences in continuous speech, using what is known as SpeechMark^®^ syllabic cluster (SC) analysis (Boyce *et al.*, 2012; Fell *et al.*, 1999; MacAuslan *et al.*, 2011). The technique, as applied in this paper, is based on automatic detection of acoustic landmarks (Stevens, *et al.*, 1992; Stevens, 2002) that characterize types of phonemes rather than specific speech sounds. This approach is distinct from ASR methods because it focuses on identifying acoustic events associated with syllables rather than attempting to recognize words.

The SpeechMark^®^ syllabic landmark detection system is an automatic, computer-based set of tools that expands work by Stevens (1985, 1998, 2002) and colleagues Liu (1996) and Howitt (2000) to detect landmarks of various sorts in the acoustic signal and group them into syllabic patterns common to English (and is available online).^1^ The acoustic landmark approach was pioneered by Stevens in the mid-1980s and leans heavily on his prior work defining the acoustic signatures of distinctive phonetic features. Stevens (1985, 1998, 2002) noted that the speech signal can be analyzed as a sequence of moments in time when articulatory movements generate abrupt change in the speech signal and, accordingly, represent areas of the signal when information density is richest and can be most easily extracted. He proposed that these time points, which he called landmarks, shape the early stages of speech perception for the running acoustic signal. In Stevens' formulation, a key point about landmarks is that they are only noted in the acoustic signal when the relevant (acoustic) distinctive features are present at a defined threshold. The assumption is that human lexical processing of the acoustic speech signal involves making yes vs no decisions about whether particular distinctive features are present. These decisions are passed up to higher levels of processing as hypotheses about the acoustic profile of the utterance. Landmarks, in essence, denote where the signal is densely packed with information vital for the initial stages of speech processing and enable the listener to formulate an initial representation of the distinctive features and consonant-vowel segmental structure of the utterance. Because more precise and accurate articulatory movements will provide more distinctive acoustic information, landmarks can be used to track the development of more or less precise motor movements for speech (Boyce *et al.*, 2011; Fell *et al.*, 1999; Fell *et al.*, 2002; Speights Atkins *et al.*, 2019). This has immediate effects on intelligibility because maximizing the information available for speech perception means that speech perception can occur in a maximally efficient manner. Missing and/or ambiguous patterns of landmarks are characteristic of less-intelligible speech because listeners will need to rely on higher-level linguistic contexts to resolve ambiguities when multiple words could be potential matches (Slifka *et al.*, 2004; Stevens *et al.*, 1992).

For purposes of identifying and classifying less precisely articulated (and, thus, less-intelligible) speech, counting the density of landmarks provides a way to quantify the difference between rich and sparse availability of lexically relevant acoustic information. We expect that the acoustic signal of maximally well-produced speech will be rich in landmarks. In contrast, if the speech signal contains fewer landmarks, then some expected acoustic information will be unavailable to the listener, and acoustic signals that represent different words may show the same pattern of landmarks.

One of the principal innovations in SpeechMark^®^ includes an algorithm to detect clusters of manner landmarks that mark differences in sonority that characterize syllable structure, described within this paper as syllabically cohesive clusters of landmarks or SCs (Fell *et al.*, 1999). The algorithm was originally developed for the purpose of tracking the development of speech motor control in infants and, accordingly, by design, lexeme- and phoneme-agnostic (Fell *et al.*, 1999). Because the system was developed for English, in the current iteration of the system, each combination of landmarks that constitutes a SC corresponds to a phonotactically well-formed syllable in English corresponding to CV, VC, CVC, etc., (Boyce *et al.*, 2013; Boyce *et al.*, 2011; Fell *et al.*, 2002). Note, however, that because landmark detection is based on the acoustic manifestation of distinctive features, the system can be adjusted to detect well-formed SCs in any language. (Note also that the use of the word “cluster” does not refer to consonant clusters but to clusters of acoustic landmarks that characterize syllables). As with landmarks themselves, whether a SC is detected depends on whether all of the distinctive features for manner in the syllable are present at a defined threshold.

In this study, we applied the SpeechMark^®^ landmark analysis system as an automated method for detecting and measuring syllabic constituents in the continuous speech signal of children. Because the approach is phoneme-agnostic, it has the advantage of being applicable when speech is unintelligible or phonetic transcription is unviable. As it is sensitive to the degree to which articulatory movements produce rich vs sparse quantities of acoustic information, it has the advantage of tracking which speech is more likely to be intelligible vs unintelligible. Further, because the output provides a quantitative measure of the number of well-formed syllables produced, it represents a method of quantifying how different groups of children may differ in speech motor control.

We focus on counts of syllabic units within continuous speech utterances spoken by preschool-age children with and without speech-related disorders [see the Instrumentation section (Sec. II D) for a detailed description of the procedures for landmark detection and their grouping into SCs]. We hypothesized that the number of syllabics predicts disordered group status. The primary focus of the investigation was to determine if automatically detected SCs can be used as a clinically relevant standard unit of measurement to identify differences in early motor development for speech when analyzing continuous speech samples of children.

## II. METHODS

### A. Ethical consideration

Ethical approval for human subject data collection was granted by the University of Cincinnati and Auburn University. Permissions were also sought from schools and the university clinic where data were collected. Parents were assured that withdrawal from the study would not harm the children in any way. Informed consent forms were signed by parents and verbal assent was obtained from the child participants. Participants' data were de-identified with codes to ensure anonymity.

### B. Dataset

The speech of 60 children, ages 3–5 years old, was analyzed using SC analysis to measure differences between groups. Of the children, 44 (age, *M* = 4.32 years old; standard deviation = 0.64) had language or speech disorder not present (SD-NP) and 16 (age, *M* = 4.14 years old; standard deviation = 0.66) were diagnosed with (SD-P, which is known to affect intelligibility but without language impairment. Speech was recorded in either of two locations—in a quiet room at a community preschool or quiet room in a university clinic. (The SpeechMark^®^ acoustical analysis tools rely on robust processing to accommodate just such environments.) Continuous speech samples were elicited for repetition from the child story book, *Brown Bear, Brown Bear, What Do You See* (Martin and Carle, 1984). From the story, 33 sentences were elicited for each subject. During the University of Cincinnati data collection, recordings were gathered using a Shure wireless system with a cardioid lavalier microphone (BLX14R/W93, Shure, Niles, IL) connected to a laptop computer. During the Auburn University data collection, recordings were gathered with a handheld ZOOM H6N recorder Zoom North America, Hauppauge, NY with MOVO cardioid lavaliere microphone (MOVO Photo, Los Angeles, CA) connected to a laptop computer. The microphone was worn by the child on a well-fitted vest. Recordings were digitally processed at a sampling rate of 44 K and 32 bit depth. Of the 1980 sentences elicited, 1952 recorded utterances were included in the analysis. Twenty-eight utterances (1.4%) were eliminated because children did not respond to elicitation probes or there was excessive environmental noise captured in the recording.

### C. Speaker classification

To be included in this study, all children were required to demonstrate normal hearing using the criterion of sound detection at 20 dB sound pressure level (SPL) for pure tones at 500, 1000, 2000, and 4000 Hz. Participants were required to exhibit age-appropriate expressive and receptive language skills on the “Clinical Evaluation of Language Fundamentals: Preschool 2” (Wiig *et al.*, 2004). Age-appropriate performance was determined by scores falling within one standard deviation of the mean (i.e., >7 scaled score or >85 standard score). Children were classified as SD-NP or SD-P using the “Clinical Assessment of Articulation and Phonology” (Secord and Donohue, 2002). Children with standard scores ≤ 85 (one standard deviation below the mean) were assigned to the SD-P group.

### D. Instrumentation

#### 1. SpeechMark^®^ matlab toolbox

The child continuous speech utterances were analyzed using version 1.5 of the SpeechMark^®^ matlab toolbox (which is available online).^1^ SpeechMark^®^ landmark detection uses the thresholds of amplitude and timing established empirically by Stevens and Liu in their work as noted above. SpeechMark^®^ acoustic landmarks come in two classes: abrupt and peak.

##### a. Abrupt-consonantal landmarks.

**FIG. 1.**
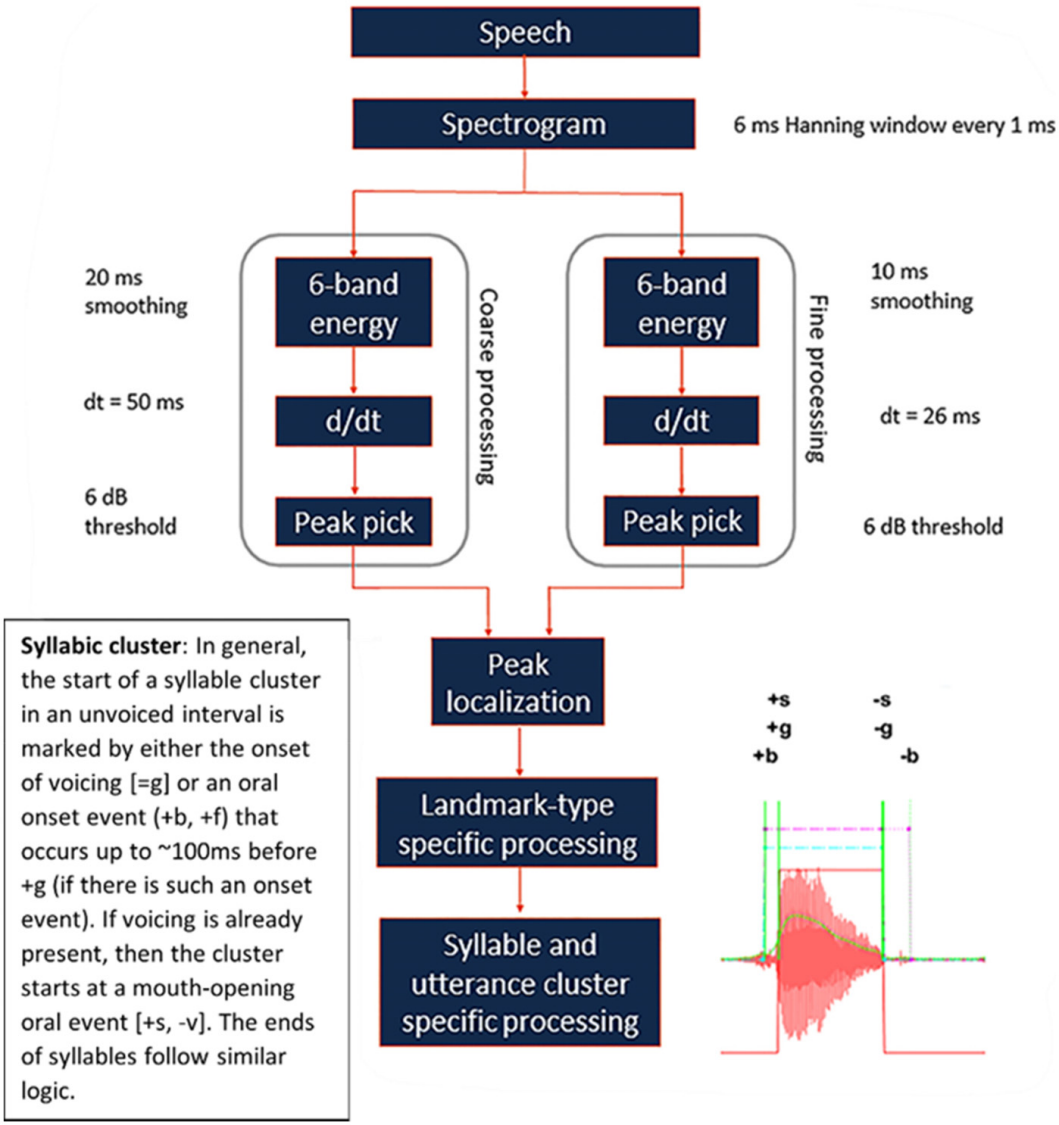
(Color online) The model of SpeechMark® landmark detection system showing landmark detection for distinctive feature-based speech recognition, adapted from Liu (1996) [J. Acoust. Soc. Am. (1996). **100**(5), 3417–3430]. Copyright 1996 Acoustical Society of America.

Abrupt or abrupt-consonantal landmarks (AC LMs, or simply LMs) have a complex specification, closely following Liu (1996). AC LMs are distinguished as either laryngeal source or vocal-tract events (Table I). AC landmark detection occurs by first computing the power in frequency bands in the spectrogram with a 6 ms Hanning window every 1 ms. The spectrogram is then divided into the six frequency bands, where the ranges are 0.15 − 0.6, 1.2 − 2.5, 1.8 − 3.0, 3.0 − 4, 4 − 6, and 6 − 8.0 kHz when specified for children. The instantaneous power is smoothed over two-time scales, approximately 25 ms (“fine”) and 50 ms (“coarse”). Coarse smoothing suppresses too-brief events, and fine smoothing allows for higher-precision placement (see Fig. 1).

**TABLE I.** Landmark types used in this study and their mnemonic labels.

	Abrupt types		
Laryngel	*g*	Glottis	Marks the beginning (+*g*) and end (−*g*) of sustained laryngeal motion near a segment of sustained periodicity (phonation) of appropriate period
	*p*	Periodicity	Marks the beginning (+*p*) and end (−*p*) of sustained periodicity of the vocal folds
	*j*	Jump	Abrupt upward or downward jump in *F*0 by at least 0.1 octave (approximately)
Vocal tract	*b*	Burst	Marks all-band frication onset or affricate/stop burst (+*b*) and the point where aspiration or frication ends (−*b*) in an unvoiced segment
	*s*	Syllabicity	Marks sonorant consonantal release, i.e., syllabic onset (+*s*) and closure, i.e., offset (−*s*) in a voiced segment
	*f*	Unvoiced frication	Marks onset (+*f*) and offset (−*f*) of simultaneous power increase at high frequencies and decrease at low frequencies outside a voiced interval
	*v*	Voiced frication	Marks onset (+*v*) and offset (−*v*) of simultaneous power increase at high frequencies and decrease at low frequencies within a voiced interval
	Peak landmark types		
Peak	*V*	Vowel	Marks a time point corresponding to local maximum of harmonic power (approximately; maximal opening of the lips)
	*F*	Continuing frication	Marks a time of maximally well-developed air turbulence as indicated by the fractal index

The algorithm localizes moments when abrupt changes and peaks in energy occur simultaneously in multiple bands and on coarse and fine time scales. An “abrupt” change occurs when power increases/decreases at the minimum of 6 dB simultaneously in the finely and coarsely smoothed contours and in at least three of the five bands (Boyce *et al.*, 2011). Simultaneity is measured to a precision of 20 ms. That is, three bands must show 6-dB increases or decreases within 20 ms of each other in the coarsely smoothed power contours, and three must show the same in the finely smoothed contours, and, finally, the coarse and fine increases or decreases must lie within 20 ms of each other. In the simplest case, power rises in all the bands on both time scales, defining a “+*b*” (unvoiced) or “+*s*” (voiced) LM. Or it may fall, likewise, “−*b*” or “−*s*,” respectively. In practice, it often happens that power rises in three or four frequency bands but stays nearly constant (to within 6 dB) in the remaining frequency bands.

A more complicated case arises for fricative-like “*f*” (unvoiced) or “*v*” (voiced) onset and offset AC LMs (not to be confused with the *F* peak LM). Here, if the power rises at high frequencies (in fewer than three bands) and simultaneously falls at lower frequencies (also in fewer than three bands), the landmark in question is labelled a “+*f*” if voiced or “+*v*” if unvoiced. Alternatively, it may do the opposite, i.e., falling at high frequencies and rising at low frequencies. These are labelled “−*f*” or “−*v*,” according to the accompanying evidence of voicing. In other words, “*b*”/“*s*” LMs always take precedence over “*f*”/“*v*.” If power rises in at least three bands, then SpeechMark^®^ identifies a “+*b*”/“+*s*.” In this case, an *f*/*v* LM is not detected even if power falls in the other bands. Similarly, when the acoustic signal shows power falling in at least three bands, SpeechMark^®^ detects “−*b*”/“−*s*.” These abrupt frication LMs may, therefore, be considered “second-chance” LMs, identifying (infrequent but clear) events that simply do not fit the *b*/*s* template.

As noted above, a key aspect of the landmark approach is that landmarks are only detected when energy change in a sufficient number of bands is sufficiently large and sufficiently simultaneous to meet the relevant threshold. This means that because thresholds are often not reached when attempts at production are ill-formed, the thresholds themselves are a particularly useful aspect of the system. Further, this approach allows for the processing of vocalizations even when they are unsuitable for speech recognition as a result of decreased speech intelligibility or the lack of words at all (as in the case of infants).

**FIG. 2.**
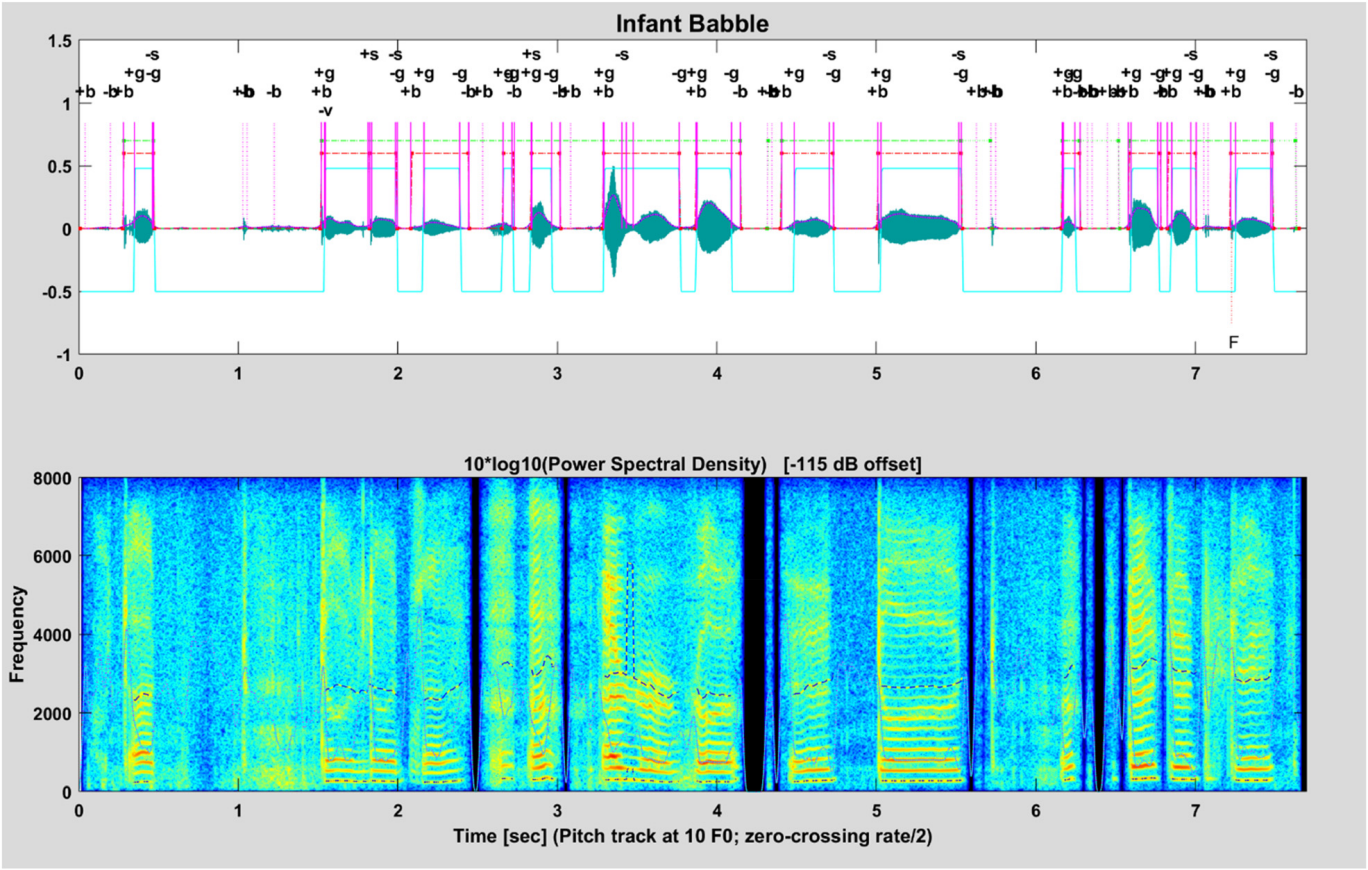
(Color online) An illustration of AC LMs and SCs that are detected in a string of infant babble.

Figure 2 shows an example of the abrupt LMs for 14 syllables of an infant babble. In contrast to peak LMs (“*F*,” shown below the waveform), SpeechMark^®^ functions draw abrupt LMs above the waveform, which are labelled by lowercase letters. The magenta vertical lines mark the moments when the landmarks occur. The horizontal red dashed lines indicate the span of each syllabic landmark cluster and, likewise, green dashed lines above those indicate the utterances. The solid cyan, dentil-like line shows the contour of voicing: positive where voiced and negative where unvoiced. The narrow-band spectrogram shows the characteristic horizontal stripes of the corresponding well-developed voicing.

#### 2. SC analysis

**FIG. 3.**
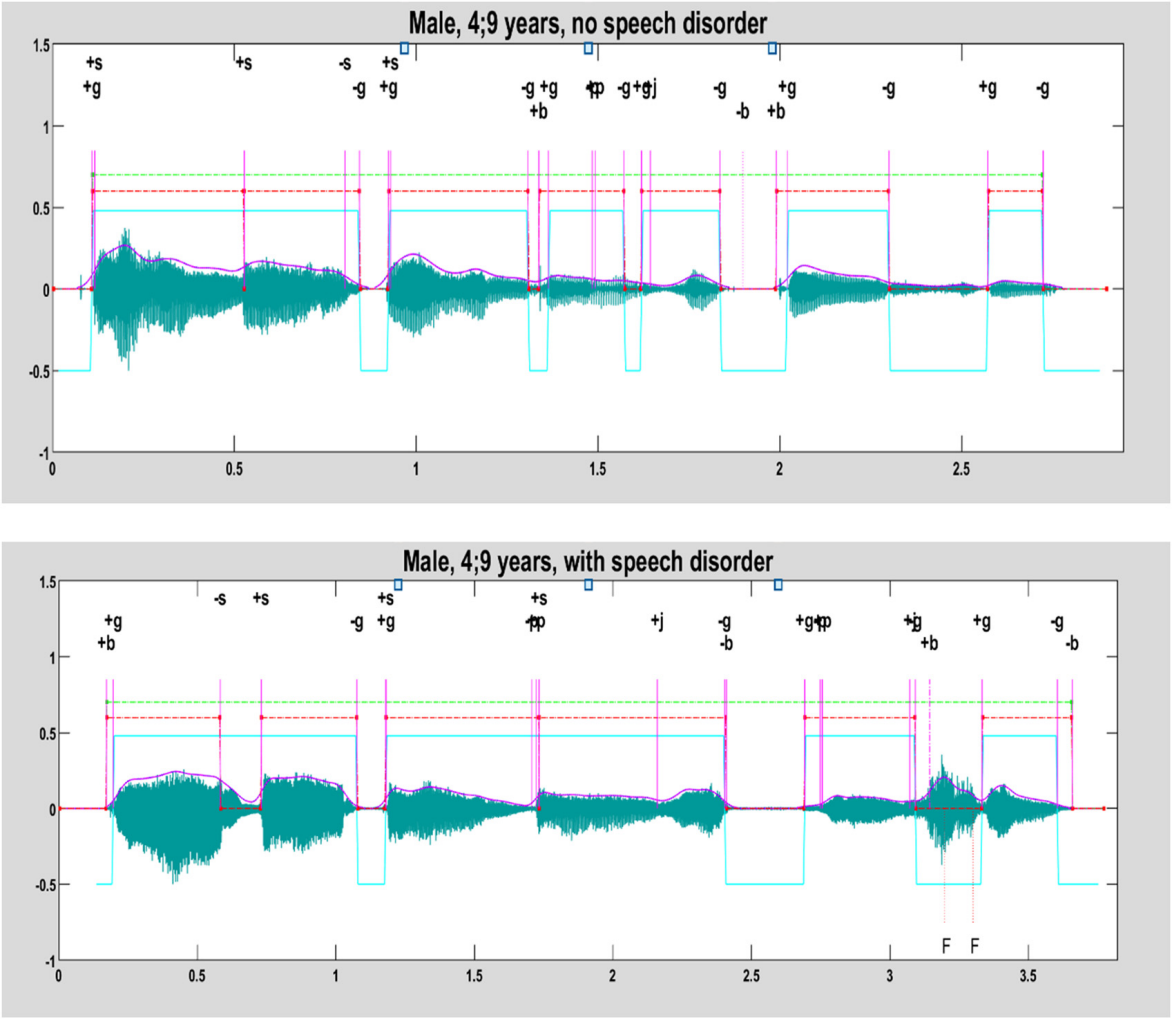
(Color online) SCs per utterance, showing the sentence “Brown bear, brown bear, what do you see?,” spoken as a single utterance by both speakers. The top figure is a waveform with plotted LMs and SCs for the speech of a child without a SD and the bottom figure is for the speech of a child of the same age with a SD. The second figure in this series shows that the utterance spoken by the child with a SD has fewer voiced intervals (four vs six) and SCs (six vs seven).

The SpeechMark® automatic SC analysis was developed from the original landmark system for the purpose of analyzing very early infant speech for emergent speech motor control. It is, therefore, intentionally meaning- and phoneme-agnostic (Fell *et al.*, 1999; Fell *et al.*, 2002). The system goes through two phases. In the first phase, the system identifies AC LMs. In the second phase, landmarks are organized into SCs (Boyce *et al.*, 2011). Consecutive abrupt oral and glottal landmarks are grouped into clusters that approximately match the shape of phototactically well-formed linguistic syllables. They are depicted by the red, dentil-like dashed lines in the Fig. 2. SCs are determined using only LMs. SC identification occurs as a process of identifying the harmonic spectrum that occurs near *g* landmarks along with abrupt oral landmarks associated with consonants. The use of acoustic landmarks allows for a more specific analysis by using vocalic and consonantal indicators of syllabicity. Syllables with a simple structure will show fewer landmarks, whereas syllables with complex structures will show more landmarks and more complex patterns of landmarks. In general, the start of a SC is marked by (1) the onset of voicing [+*g*] (purely laryngeal), (2) an oral onset event [+*b*,+*f*] that occurs up to ∼100 ms before the onset of voicing [+*g*], or (3) with an oral LM release [+*s*,−*v*] that occurs during voicing but more than ∼100 ms after the start of the previous SC. SCs occur within utterance clusters and intervals occur when there is silence that is longer than ∼350 ms. (The green lines in Fig. 2 mark multiple utterances in a string of infant babble while Fig. 3 shows SCs within a single utterance.)

SC analysis allows for the later analysis of differences in the inventory of landmarks within the SCs. Recall that the detection of abrupt landmarks is based on thresholding—when a syllable is produced with articulatory movements that are strong and timed appropriately, the acoustic signatures will meet the threshold, and the full set of landmarks will be detected. When the same syllable is spoken with weaker and/or mistimed articulatory movements, the landmark thresholds will not always be reached, and fewer landmarks will be detected. Thus, different SC groupings will be detected when a string of intended syllables is produced in (1) its canonical form (e.g., CCVC), (2) a less complex form (CVC), or (3) a more lenited (i.e., softened consonant) form: /*t*/ becomes /*ɵ*/, /*s*/ becomes /*f*/, etc. For example, the sequences “aah,” “bah,” and “bat” consist of V, CV, and CVC syllables, respectively. If produced canonically, they would be distinguished from one another as +*g*−*g*, +*b*+*g*+*s*−*g*, and +*b*+*g*+*s*–*g*+*b*−*b*. At the same time, unclearly articulated sequences may exhibit similar SC shape (e.g., bat may present as +*b* + *g*+*s*−*g* or bah). Similarly, unclearly articulated sequences may be absorbed into neighboring SCs. It is a feature of the SpeechMark^®^ system that words are represented as they are produced rather than according to their nominal phoneme content, and many words with similar syllabic shape will produce identical sequences of landmarks.

It is known that young children and children with SD-P are characterized by having less precise articulation and longer speaking duration (Flipsen, 2002; Kim and Stoel-Gammon, 2010) than their typically speaking peers. This relationship is illustrated in Fig. 3, which displays the landmark sequences and SC groupings for productions of the utterance “Brown bear, brown bear, what do you see?” by preschool-age children with SD-NP vs SD-P. Differences in the groupings of landmarks within SCs, the number of SCs detected, and speech duration are noted. Figure 3 shows that the utterance spoken by the child with SD-P has longer duration and more landmark dispersion, resulting in different groupings of SCs.

#### 3. Data analysis

We used a two-step approach for the generation and analysis of the SC measures. First, we processed the acoustic landmarks for each utterance to obtain the sequence of landmarks and then grouped them into SCs. Further, we computed speech rate (SCs per unit time) and duration (time in seconds to produce the target sentence from start of the first SC to end of the last SC).

#### 4. Statistical analysis

The primary goal of the study was to examine whether speakers with SD-NP demonstrate a higher frequency of SCs compared to the SD-P group and determine if other SpeechMark^®^ measures could be useful predictors. SCs were chosen as the primary focus as a standard measure that could be used across the developmental years, however, we wanted to comprehensively evaluate each of the seven automated SpeechMark^®^ measures. To do this, we first performed a four-factor regression analysis (see below) to evaluate the robustness of each of the seven automated measures produced by SpeechMark^®^: number of utterances (#Utts), phrase duration (Dur), number of landmarks (#LMs), number of syllabic LM clusters (#SCs), LMs per SC, SCs per utterance, and SC rate. SC rate is defined as SCs/duration.

With speech group as our central point of interest, we employed a generalized linear model (GLM) using a negative binomial distribution to assess the number of SCs produced by children with and without SD-P. All statistical analyses were performed using RStudio version 2024.04.1 (Posit team, 2024) with MASS (Ripley *et al.*, 2024) and lme4 (Bates *et al.*, 2024) packages. Model parameters included speech group, segment duration, age, and speech group × age. This model choice is particularly suited for count data, such as SCs, and adeptly handles over-dispersion—a scenario in which the variance exceeds the mean. Overdispersion in child speech count data, such as the number of syllables, can be attributed to factors like variability across developmental stages, context-dependent speech complexity, and unaccounted influences, including cognitive development or socio-economic status. GLMs are also well-suited to handle varying sample sizes across groups, however, one common approach to addressing unequal group sizes is to weight the observations such that groups with fewer observations have a greater influence on the regression outcome relative to their size. This can help mitigate the bias introduced by larger groups dominating the results. Although the unequal group sizes should not impact the analysis due to the robustness of the model, we nevertheless included the use of weights based on the group sizes.

To quantify the relationship between the groups and the frequency of SCs, we introduce the incidence rate ratio (IRR) as a key measure. The concept of the incidence rate, predominantly used in epidemiological research, is adapted to describe the occurrence frequency of particular events, such as diseases or conditions, within a specific population over a designated timeframe. The IRR in this case offers a comparative metric of the incidence rates of SCs between the SD-NP and SD-P groups, providing insights into the relative frequency of SC occurrence in each group. The inclusion of IRR in our analysis is pivotal in understanding the proportional differences in SC frequencies between the two groups under study. Statistically significant differences between the groups could serve as a diagnostic indicator of SD when evaluating continuous speech.

## III. RESULTS

### A. Descriptive statistics

The descriptive statistics are displayed in Table II.

**TABLE II.** Descriptive statistics. *N*, utterances per group; mean (standard deviation), *n* (%).

	SD-NP	SD-P
Characteristics	*N* = 441	*N* = 161
Age	4.32 (0.64)	4.14 90.65)
Language variation (%)		
Midland	23 (52%)	9 (56%)
Southern	21 (48%)	7 (44%)
LMs (*n*)	18.37 (2.24)	18.99 (2.43)
SCs (*n*)	5.84 (0.46)	5.93 (0.61)
Utterance per sentence	1.28 (0.28)	1.50 (0.52)
SCs per utterance	5.01 (0.72)	4.59 (1.19)
Speech duration (s)	2.14 (0.41)	2.25 (0.48)
Speech rate (SCs/s)	2.66 (0.48)	2.47 (0.65)

### B. Regression analyses

#### 1. Four-factor regression analysis of all SpeechMark^®^ measures

Our focus was to identify one or more objective measures that could be clinically useful, although there are admittedly too few subjects, particularly SD-P, to quantify this here and is not within the scope of this study. We began by removing 28 test items for which the child has produced no usable recording: 17 SD-NP and 11 SD-P. Only 1 child, a SD-NP, produced 4 such items among the 33 in the set; and 1, a SD-P, produced 3 such items. Given the small fractions, we kept these subjects in the analysis to follow.

This left 1952 individual observations remaining. For each observation, we ran automated SpeechMark^®^ analyses to produce the seven measures: number of utterances (#Utts), phrase duration (Dur), number of landmarks (#LMs), number of syllabic LM clusters (#SCs), LMs per SC, SCs per utterance, and SC rate, which is defined as SCs/duration.

For each measure, we performed four-factor linear regressions: on speech group (SG), dialect group (DG), phrase length (len, short or long), and age. We ignored single-factor analyses: Our focus places speech group at the center and, realistically, we could not *a priori* ignore its potential interactions with the other factors. Because of the large number of possible coefficients, 15 per measure, we set a statistical significance threshold of 0.01. As it happens, LM/SC was not significant in any way (beyond its grand mean, of course). Briefly, the significant results for each measure are noted in Table III.

**TABLE III.** Four-factor regression analyses on SG, DG, phrase length (len, short or long), and age. *, significant at *p* ≪ 0.001.

Measures	Results
#Utts	Len(*), SG × len(*), age × len(*) DG × len, SG × age × len, and Age × DG × len
Duration	Len(*) and age × len(*).
#SCs	SG, len(*), SG × age
#LMs	Len(*) alone
SC per Utt	Age × len(*), DG × len, age × DG × len
SC rate	Len(*), age × len.

With our stated focus, #Utts and #SCs are the measures of interest. They have the only significant sensitivity to speech group. Moreover, both are sensitive to interactions between SG and age. Conveniently, both are counts. However, #Utts is a more “blunt instrument” than #SCs because of this very quantization: Typical values for #Utts are 1–2 for short phrases and 3–6 for long phrases. In contrast, #SCs is approximately four times higher. Finally, for the numerically insatiable, *F*(15,1936) = 33.8 and *R*^2^ = 0.201 for #Utts, but 237.1 and 0.648 for #SCs, again, favoring the latter measure.

In summary, the number of SCs appears to be the best choice of clinically focused measure among the seven automated measures considered. Given its nature as a count, we analyzed SCs further with a negative binomial regression.

#### 2. Negative binomial regression

Table IV presents the coefficients from a negative binomial regression model, detailing estimates, standard errors, *z*-values, and *p*-values [Pr(>|*z*|)] for each variable. The intercept has a significant positive estimate of 0.88948 with a small standard error, indicating a strong effect when all other variables are at zero. Speech group1 (SD-P) is associated with a significant decrease in the SCs as shown by its negative coefficient of –0.20757, which is statistically significant (*p* = 0.0229). The variable seg.duration (longer duration) has a positive coefficient of 0.28672, which is significant (*p* < 0.001), suggesting a substantial increase in the SCs with each unit increase in segment duration. Age is also significantly associated with SCs (*p* ≤ 0.001). The interaction term speech group1 × age has a positive estimate of 0.04328. Age-related changes in syllable production are significant in the SD-P group (*p* = 0.0429), indicating that the effect of speech group1 changes with age.

**TABLE IV.** Negative binomial coefficients. Significant codes, ^***^, 0.001; ^**^, 0.01; ^*^, 0.05.

	Estimate	Standard error	*z* value	Pr(>|*z*|)
(Intercept)	0.88948	0.0551	16.121	<0.001^***^
Speech group1 (SD-P)	−0.20757	0.0911	−2.277	0.0229^*^
Age	0.05044	0.0119	4.229	<0.001^***^
Seg.duration	0.28672	0.00533	53.701	<0.001^***^
Speech group1: Age	0.04328	0.02136	2.026	0.0429^*^

#### 3. Incidence rate ratio

Table V displays the IRR estimate for the respective variable, along with the 95% confidence interval (CI). For children in speech group1 (SD-P), the SC rate is 0.8116 times the rate, indicating significantly fewer SCs compared to the reference group (*p* = 0.0137). For age, the SC count increases by a factor of 1.0485, indicating a significant effect (*p* = 0.0112). This suggests an additional year of age results in approximately 1.05 times more syllables. Longer segment durations (seg_duration) are significantly associated with higher SCs (*p* ≤ 0.001), indicating that for every unit increase in segment duration, the syllable count increases by a factor of 1.3175. The interaction term speech group1 and age suggests that for every 1-yr increase in age within the SD-P category, the SC rate is multiplied by 1.0451. The SC count increases more significantly (*p* = 0.0251), with each additional year compared to the SD-NP reference group.

**TABLE V.** Negative binomial regression results predicting cumulative syllable count.

Predictor	Estimate	IRR	Lower CI	Upper CI	95% CI	*p*-value
(Intercept)	0.9296	2.5335	2.2246	2.8853	[2.22,2.89]	<0.001^***^
Speech group1 (SD-P)	−0.2088	0.8116	0.6875	0.9580	[0.69,0.96]	0.0137^*^
Age	0.0474	1.0485	1.0188	1.0791	[1.02,1.08]	0.0012^**^
Seg_duration	0.2757	1.3175	1.3042	1.3309	[1.30,1.33]	<0.001^***^
Speech group1:age	0.0441	1.0451	1.0056	1.0862	[1.01,1.09]	0.0251^*^

## IV. DISCUSSION

### A. Large-scale analysis of child continuous speech

Multiple studies using phoneme-based transcription have shown that the ability to produce well-formed syllables is a reliable indicator of speech production mastery in continuous speech recordings of child speech (Fell *et al.*, 1999; Fell *et al.*, 2002; Flipsen, 2006b; Masso *et al.*, 2016; Oller *et al.*, 2010; Stoel-Gammon, 2010; Yaruss, 2000), but the laborious and time-intensive nature of the approach has limited its practical use. Automated procedures have added convenient and fully objective acoustic analysis approaches with increasing success (Boyce *et al.*, 2011; de Jong and Wempe, 2009; Howitt, 2000; Mermelstein, 1975; Seshadri and Rasanen, 2019; Villing, 2005), although only a few have focused on the use in controlled continuous speech samples that can be used for the identification of children with atypical speech development. Our work is aimed at establishing an automated method for large-scale analysis of continuous, not necessarily intelligible, speech samples spoken by young children with an eye to identifying the degree to which acoustic information important for speech perception is embedded in the acoustic signal.

This study introduces SpeechMark^®^ automatic analysis of syllabic landmark clusters, a computational method that correlates with intelligibility (Boyce *et al.*, 2013) for analyzing continuous speech in children with SD-P and SD-NP. The SpeechMark^®^ approach has several advantages. It avoids the problems of phoneme-based approaches in that speech analyzed need not be intelligible in terms of intended words. At the same time, the fact that it is automated allows for fast results on longer, continuous speech samples that are known to be more reliable measures of child speech production mastery.

Our findings demonstrated that children with SD-P produced fewer SCs than children with SD-NP, aligning with previous research observing syllable production in young children with speech-related conditions (Fell *et al.*, 2002; Flipsen, 2006b; Yaruss, 2000). Furthermore, the interaction with age revealed that the relationship between age and trajectory of increasing SC production is different for children with SD-P and children with SD-NP, where the rate of SC production increases more and more rapidly for children with SD-NP. This may be because more stable syllable count development at different ages in the SD-NP group, whereas children with SD-P are gradually catching up over time, as indicated by the significant 4% increase in SC per year of additional age. A longitudinal study with more children would allow us to model growth curves to evaluate the slope of this developmental trajectory in both groups.

The observed interaction between SCs and age underscores an expected maturation effect, indicating that while children with SD-P initially produce SCs at a slower rate, this rate increases with age.

### B. Limitations

One limitation of the current study is that we did not include direct measures of speech severity in the statistical models to identify the relationship between speech severity and SC detection. As stated above, this study sought to analyze child speech samples with a controlled unit of measurement that would not require phonetic transcription of unintelligible segments and would reflect a more direct measure of articulatory control in a heterogenous group of preschool-age speakers. Further investigations are warranted to understand the relationship between acoustic landmarks detected and their subsequent groupings effect on speech accuracy and intelligibility.

A minor limitation is the small size of the different dialect groups. Our result of no statistically significant difference was somewhat expected because the dialects at issue do not differ very much in the acoustic characteristics measured by SpeechMark^®^, but the small size of the groups necessitates a tentative conclusion. We also approached the interpretation of the age effects with caution because of the sample size and lack of equal numbers of children with and without SD in each age group. Another limitation to note is that these results are specific to the items elicited and future work could include different prompts with a larger group of children to determine if there are item-level effects.

### C. Conclusions

Building on Stevens' landmark theory, our study delved into continuous speech patterns among preschool-age children with SDs, unveiling marked differences in SC production compared to their typically developing counterparts. Departing from traditional approaches in automatic speech recognition, our focus on acoustic landmarks and their configurations as speech biomarkers marks a pivotal innovation. This phoneme-agnostic methodology addresses the prevalent challenges of reduced intelligibility and heightened variability in young children's speech.

Automating large-scale continuous speech data analysis carries significant clinical relevance. Conventional speech pattern capture relies heavily on subjective phonetic transcription, intertwined with human speech perception, a challenge when decoding signals with missing information, as elucidated by Stevens' insights. Children with SDs exhibit reduced acoustic richness, potentially complicating listener comprehension. Our landmark-based approach could quantitatively gauge a child's speech production and developmental trajectory, offering ecologically valid insights into everyday speech function.

Our findings underscore the influence of SD status and age on speech patterns, presenting a transformative potential for clinical applications. Automated tools, inspired by Stevens' principles, hold promise in objectively assessing speech characteristics among children with SDs. The identification of speech-specific markers in continuous speech samples has groundbreaking implications, revolutionizing diagnostic precision and customized interventions for improved speech outcomes and overall communication skills in this demographic.

The innovation of automated SC detection represents a breakthrough in analyzing continuous speech among young children. Demonstrating the effectiveness of SpeechMark^®^ landmark-based technology in detecting speech-performance differences reinforces the use of SCs as an objective measurement unit, even in preschool-age children with reduced speech intelligibility caused by disorders. Leveraging these markers could fundamentally redefine clinical practices, enabling early identification and tailored therapies to enhance the communication abilities of children affected by SDs.

## Data Availability

The data that support the findings of this study are available from the corresponding author upon reasonable request.

## Footnotes

^1^
See https://speechmrk.com/ (Last viewed August 12, 2024).
